# Supplementary material for: Evaluation of a Push–Pull Strategy for Spotted-Wing Drosophila Management in Highbush Blueberry
Source: Insects. 2024 Jan 10;15(1):47. doi: 10.3390/insects15010047 (PMC10816925; doi:10.3390/insects15010047)
Supplement: Supplementary file 1 [file insects-15-00047-s001.zip › insects-2784494-supplementary.pdf]

Supplementary Materials

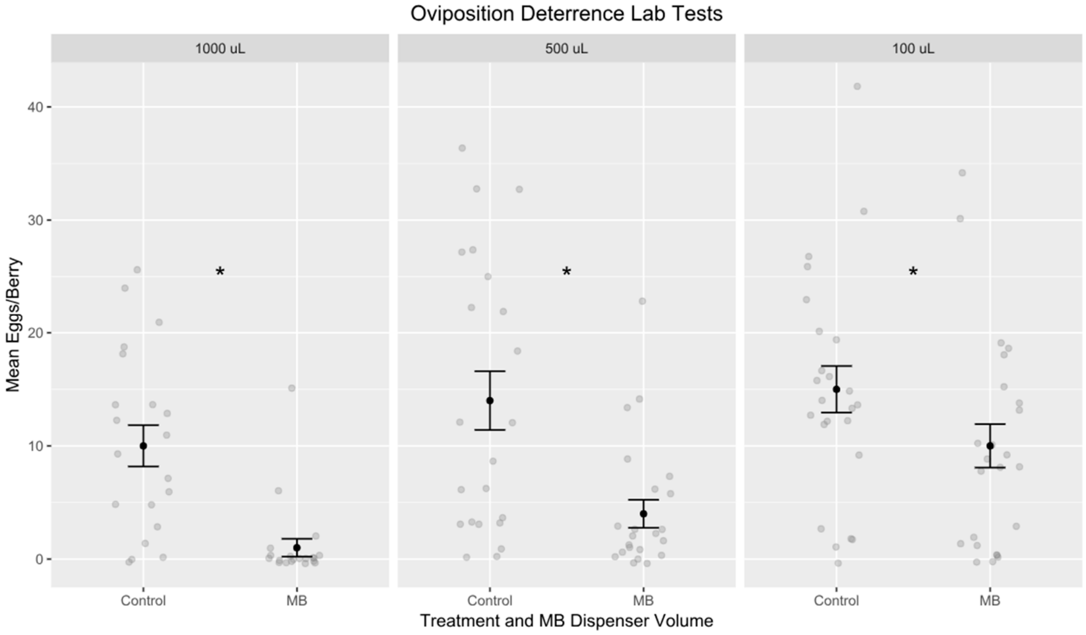

Figure S1. Black points and brackets represent overall means +/- 1 SE while grey points represent the mean for each replicate. An asterisk indicates significant differences between treatments in a set of tests. P < 0.0001 in all cases. 1000 uL dispensers emitted ~15 mg MB/h, 500 uL dispensers emitted ~11 mg MB/h, and 100 uL dispensers emitted ~7 mg MB/h. For 1000 uL dispenser tests N = 20, for 500 uL tests N = 22, for 100 uL tests N = 24.

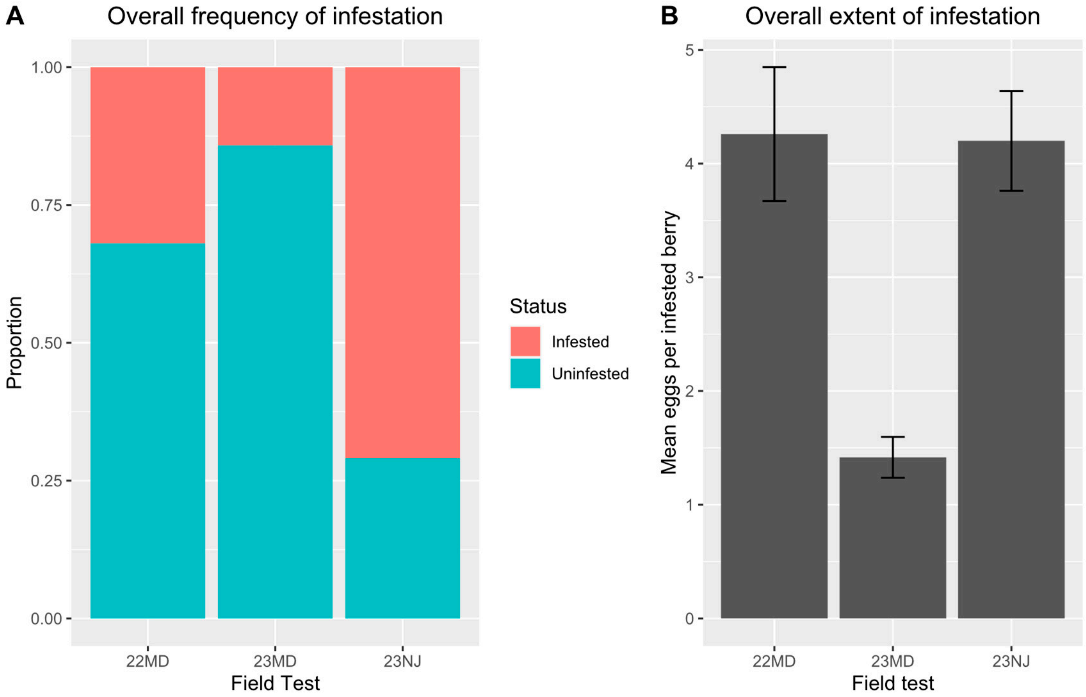

Figure S2. A) The overall proportions of berry infestation by SWD in each field test. B) The overall mean number of eggs per infested berry in each field test. 22MD: 2022 field test at the Maryland site, 23MD: 2023 field test at the Maryland site, 23NJ: 2023 field test at the New Jersey site. Frequency of infestation for each test: 22MD, 32%; 23MD, 14%; 23NJ 71%. The 22MD test had a mean of 3.77

eggs/infested berry, the 23MD test had a mean of 1.21 eggs/infested berry, and the 23NJ test had a mean of 4.19 eggs/infested berry. 22MD N = 99, 23MD N = 93, 23NJ N = 69.

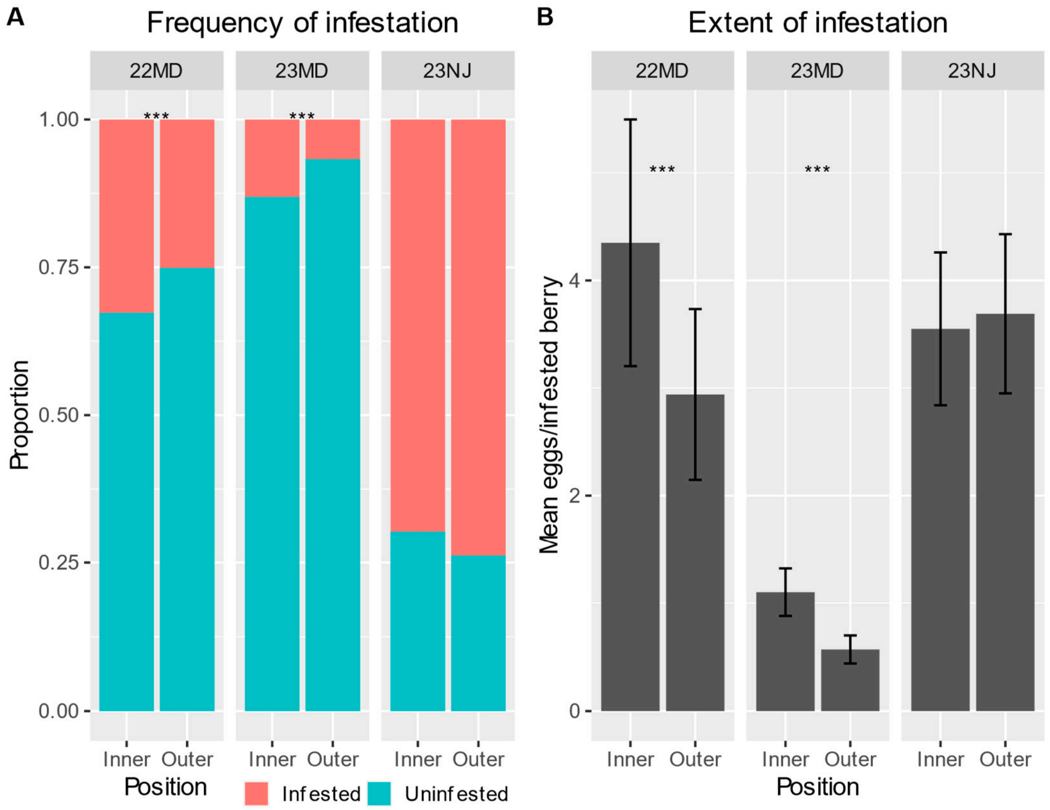

Figure S3. In panels assigned a triple asterisk (***), differences due to position are highly significant at α = 0.01. Statistics for pairwise comparisons are presented in Table S1. A) The proportion of blueberries infested with at least one SWD egg on inner- or outer-canopy branches for all field tests combined and individually. Proportions are estimated by the logistic regression models. B) The mean number of SWD eggs in infested blueberries on inner- or outer-canopy branches for all field tests combined and individually. Means are estimated by the generalized linear models.

Table S1. Pairwise comparisons of the estimated marginal means from the models for the frequency and extent of infestation as responses to treatment in all field tests. Comparisons were only made for tests in which treatment had at least a marginally significant effect (Table 3). P values are adjusted for multiple comparisons by Tukey's method within each response. Significance codes: double asterisk (**), significant at $\alpha = 0.05$; triple asterisk (***), highly significant at $\alpha = 0.01$.

Field Test	Response	Factor	Contrast	Z-ratio	P value
2022 MD	Frequency of infestation	Position	Inner, Outer	2.742	0.0061***
	Extent of infestation	Treatment	Control, Push	0.664	0.9106
			Control, Pull	-2.172	0.1312
			Control, Push-Pull	0.551	0.9464
			Push, Pull	-2.68	0.037**
			Push, Push-Pull	-0.104	0.9996
			Pull, Push-Pull	2.572	0.0497**
		Position	Inner, Outer	3.695	0.0002***
2023 MD	Frequency of infestation	Position	Inner, Outer	5.151	< 0.0001***
	Extent of infestation	Position	Inner, Outer	3.580	0.0003***
2023 NJ	Frequency of infestation	Treatment	Control, Push	6.875	< 0.0001***
			Control, Pull	1.685	0.3316
			Control, Push-Pull	4.842	< 0.0001***
			Push, Pull	-5.575	< 0.0001***
			Push, Push-Pull	-2.117	0.1476
			Pull, Push-Pull	3.384	0.004***
		Position	Inner, Outer	-1.514	0.1299
	Extent of infestation	Treatment	Control, Push	5.256	< 0.0001***
			Control, Pull	3.3685	0.0013***
			Control, Push-Pull	4.883	< 0.0001***
			Push, Pull	-1.565	0.3988
			Push, Push-Pull	-0.104	0.9996
			Pull, Push-Pull	1.395	0.5026
